# Genetic makeup of Shiga toxin-producing ***Escherichia coli*** in relation to clinical symptoms and duration of shedding: a microarray analysis of isolates from Swedish children

**DOI:** 10.1007/s10096-017-2950-7

**Published:** 2017-04-19

**Authors:** A. Matussek, C. Jernberg, I.-M. Einemo, S. Monecke, R. Ehricht, I. Engelmann, S. Löfgren, S. Mernelius

**Affiliations:** 1 Karolinska University Laboratory, Stockholm, Sweden; 2 Microbiology Laboratory, Division of Medical Diagnostics, Region Jönköping County, Jönköping, Sweden; 3 Public Health Agency of Sweden, Solna, Sweden; 4 Department of Communicable Disease Control, Region Jönköping County, Jönköping, Sweden; 5 Alere Technologies GmbH, Jena, Germany; 6 Institute for Medical Microbiology, Virology and Hygiene, Technische Universität Dresden, Dresden, Germany; 7 InfectoGnostics Research Campus, Jena, Germany

## Abstract

Shiga toxin (Stx)-producing *Escherichia coli* (STECs) cause non-bloody diarrhea, hemorrhagic colitis, and hemolytic uremic syndrome, and are the primary cause of acute renal failure in children worldwide. This study investigated the correlation of genetic makeup of STEC strains as revealed by DNA microarray to clinical symptoms and the duration of STEC shedding. All STEC isolated (*n* = 96) from patients <10 years of age in Jönköping County, Sweden from 2003 to 2015 were included. Isolates were characterized by DNA microarray, including almost 280 genes. Clinical data were collected through a questionnaire and by reviewing medical records. Of the 96 virulence genes (including *stx*) in the microarray, 62 genes were present in at least one isolate. Statistically significant differences in prevalence were observed for 21 genes when comparing patients with bloody diarrhea (BD) and with non-bloody stool (18 of 21 associated with BD). Most genes encode toxins (e.g., *stx2* alleles, *astA*, *toxB*), adhesion factors (i.e. *espB*_O157, *tir*, *eae*), or secretion factors (e.g., *espA*, *espF*, *espJ*, *etpD*, *nleA*, *nleB*, *nleC*, *tccP*). Seven genes were associated with prolonged *stx* shedding; the presence of three genes (*lpfA*, *senB*, and *stx1*) and the absence of four genes (*espB_O157*, *espF*, *astA*, and *intI1*). We found STEC genes that might predict severe disease outcome already at diagnosis. This can be used to develop diagnostic tools for risk assessment of disease outcome. Furthermore, genes associated with the duration of *stx* shedding were detected, enabling a possible better prediction of length of STEC carriage after infection.

## Introduction

Shiga toxin (Stx)-producing *Escherichia coli* (STEC) are causative pathogens of non-bloody diarrhea (NBD), bloody diarrhea (BD), hemorrhagic colitis (HC), and hemolytic uremic syndrome (HUS), and are the most common cause of acute renal failure in children worldwide [1–4]. There is a large variety of STEC circulating within cattle; however, only a subset of them seem to cause disease in humans. Human STEC isolates are also designated enterohemorrhagic *E. coli* (EHEC), and O157:H7 is actually the predominant serotype responsible for outbreaks worldwide [5, 6]. The main focus for diagnostics has therefore focused on EHEC O157; however, also non-O157 serotypes, such as O26, O103, O111, and O145, contribute significantly to cases of diarrhea, HC, and HUS [6]. In 2011, a large outbreak of *E. coli* O104:H4 in Germany led to HUS in more than 800 patients, and 53 deaths [7–9]. It is recommended that samples from suspected STEC-infected patients should be tested as soon as possible after onset of symptoms [10]. Also, rapid STEC detection is important in outbreak management and patient treatment, including prompt parenteral hydration, monitoring for development of severe disease, and avoidance of antibiotics and antidiarrheal agents, which can exacerbate disease [11].

Stx is the most important virulence factor in EHEC/STEC. It is divided into two major types, Stx1 and Stx2, where Stx2 is responsible for the most cases with severe symptoms. Stx1 and Stx2 are further categorized into several subtypes; Stx1 consists of three subtypes, *stx1a, stx1c*, and *stx1d*, whereas Stx2 is composed of seven subtypes; *stx2a, stx2b, stx2c, stx2d, stx2e, stx2f*, and *stx2g* [12]. The Stxs are not exclusively responsible for pathogenesis of STEC; several other virulence factors encoded by genes carried on mobile genetic elements also play a role [13, 14]. A major virulence factor is the outer membrane protein intimin, encoded by *eae*. Intimin is thought to be the determinant of the formation of attaching and effacing (A/E) lesions. Additional virulence markers are enterohemolysin and, in strains lacking *eae*, an autoagglutinating adhesin (Saa). Furthermore, Ferdous et al. recently showed that isolates with virulence genes encoding type III secretion proteins and adhesins were associated with HC, and that these isolates were from diverse phylogenetic backgrounds [15]. In addition, de Boer et al. proposed a diagnostic algorithm applied directly on stool samples of patients presenting with gastrointestinal symptoms to assess the public health risk of STEC infection [16].

A variety of methods have been used to classify STEC. Karmali et al. introduced seropathotypes to assess the pathogenic potential of STEC on the basis of their reported frequencies in human illness [17]. In addition, specific genetic lineages of STEC, such as the clade 8 of EHEC O157:H7, apparently are more prone to cause severe disease [18]. The analysis of single nucleotide polymorphisms (SNPs), also useful for outbreak investigations, can resolve closely related bacterial genotypes, and contribute to associations between bacterial genetic makeup and disease severity [18].

There are reports showing long-term STEC shedding, a long time after symptoms are resolved. The median duration of shedding has been shown to be 20 days; however, some patients were *stx* PCR-positive for up to 9 months [19, 20]. This can have major implications for families. For instance, in Sweden there is a legal requirement for at least one *stx*-negative stool sample before children are allowed to return to kindergarten. A recent study could not find an association between duration of shedding and either *stx* type nor the presence of intimin [19]. The possibility to predict duration of shedding based on STEC features would enable optimized infection control measures.

DNA-microarray-based genotyping of STEC facilitates the simultaneous detection of hundreds of different genes including virulence, typing, and resistance markers. This information can also be used to assign isolates to different lineages and determine the genetic relationship between them, comparable with analysis of SNPs mentioned above and to discriminate between different *stx* subtypes [21, 22].

The aim of this study was to characterize STEC isolated from children in Region Jönköping County, Sweden from May 2003 through January 2015 by microarray analysis, and correlate genetic makeup of strains to clinical symptoms and duration of STEC shedding. This could facilitate an assessment of the public health risk of strains when dealing with infected patients, optimized treatment regimens, and control measure guidelines.

## Materials and methods

### Patients and isolation of bacteria

From the 215 children <10 years in Jönköping County (approximately 330,000 inhabitants, served by three hospitals and 46 health care centers) from May 2003 through January 2015 with PCR-positive STEC samples, all cultivable primary STEC isolates (*n* = 96) were included. STEC detection was done by detection of *stx* by real-time PCR on suspensions of overnight cultures on blood agar plates [23]. PCR positive specimens were sent to the Karolinska University Laboratory, Stockholm, Sweden for confirmatory testing and isolation of STEC according to methods described by Svenungsson B. et al. [24]. Patients were sampled weekly until they were *stx*-PCR-negative, and the duration of *stx* shedding was defined as the time from the first positive sample to the first negative sample, as previously described [19].

### Isolate characterization

In Sweden, all STEC isolates are submitted to the The Public Health Agency of Sweden for confirmation and further typing as part of the national microbial surveillance program. In the Agency, isolates were serogrouped (with regard to O-type) by agglutination in micro titer plates using antisera (SSI Diagnostica, Copenhagen, Denmark).

All primary STEC isolates were subjected to DNA-microarray analysis with the E. coli PanType AS-2 Kit, E. coli SeroGenoTyping AS-1 Kit, and ShigaToxType AS-2 Kit according to manufacturer’s instructions (http://alere-technologies.com/en/products/lab-solutions/shigatoxin.html, http://alere-technologies.com/en/products/lab-solutions/e-coli/e-coli-serogenotyping-kit.html, http://alere-technologies.com/en/products/lab-solutions/e-coli/e-coli-pantype-kit.html). The three different microarray-based assays performed for each isolate included the determination of the serogenotype and the *stx* allele/subtype [12, 22], as well as the detection of antibiotic resistance genes, and a large number of other virulence genes [22, 25, 26].

Clinical data were collected from all patients through a questionnaire and by reviewing medical records (Table 1). Clinical symptoms (diarrhea, bloody diarrhea, abdominal pains, vomiting, fever, and HUS) were concluded in a clinical symptom score ranging from 0 to 6, where 6 corresponded to the most severe presentation. Criteria for HUS included three primary symptoms: hemolytic anemia with fragmentocytes, low platelet count, and acute renal failure with a creatinine above the age-specific reference range.

**Table 1:** Patient data and clinical symptoms for all cases of STEC infection as well as patients with bloody diarrhea (BD) and with non-bloody stool (NBS)

	All cases	BD	NBS	*P*-value^a^
No. (%)	96	17 (18)	79 (82)	
Median age of patients (interquartil range)	2 (1–5)	2 (1–4)	3 (1–6)	0.3
Median length of carriage, in days (interquartil range)	24 (14–48)	22 (11–47)	24 (14–48)	0.6
Aquired bacteria abroad (%)	41 (43)	5 (29)	36 (46)	0.2
Diarrhea (%)	79 (82)	17 (100)	62 (78)	0.03*
Fever (%)	18 (19)	8 (47)	10 (13)	0.003*
HUS (%)	1 (1)	1 (6)	0 (0)	0.2
Vomiting (%)	12 (13)	2 (12)	10 (13)	1.0

^a^comparing BD to NBS

*statistically significant difference

### Statistical analyses

*χ*2 and Fisher’s exact test were done for comparing categorical data, using Statistica 12 (StatSoft, Inc. Tulsa, OK) and VassarStats (http://vassarstats.net). Mann–Whitney *U* test was used for comparing continuous data, using Statistica (Statsoft, Inc.). Kaplan–Meier survival analyses were done using Statistica 12 (Statsoft, Inc.). Significant findings from univariate logistic regression were further explored by multiple logistic regression models, using a forward stepwise approach (Statistica v.13.1, Statsoft, Inc.). Due to the small sample size, subsets of no more than ten genes were analyzed in each multiple logistic regression model. *P* < 0.05 was considered statistically significant in all statistical analyses. A minimum spanning tree (MST) based on the 62 virulence genes present in at least one isolate was constructed using BioNumerics v. 6.1 (Applied Maths, Sint-Martens-Latem, Belgium).

## Results

### Patient data, *stx* subtypes, and serogenotype distribution

Patient data and clinical symptoms are presented in Table 1. In total, 79 patients suffered from diarrhea, and 17 of these developed BD (one case with BD also developed HUS). Fourteen patients were free of symptoms, most of which were sampled for contact tracing and others due to other symptoms other than the six included in the clinical symptom score.

Seven different *stx* subtypes were found among the isolates, *stx1a* being most predominant (*n* = 31,;32%). Nineteen isolates presented a combination of two or more *stx* subtypes, *stx2a*/*stx2c* being the most predominant combination (*n* = 7; 7%). The BD cases were caused by *stx1a* (*n* = 5), *stx2a* (*n* = 9) and *stx2c* (*n* = 8) positive isolates, and the HUS case by a *stx2a* and *stx2c* positive isolate (Table 2).

**Table 2:** Number of isolates with the different stx subtypes

*stx* type	No.		
	Total	BD	NBS
*stx1a*	31	3	28
*stx1* ^*a*^	14	1	13
*stx2c*	8	1	7
*stx2a*	7	4	3
*stx2a/stx2c*	7	5	2
*stx2* ^*a*^	6	1	5
*stx1c*	4		4
*stx2b*	4		4
*stx1* ^*a*^ */stx2b*	3		3
*stx1* ^*a*^ */stx2* ^*a*^	2		2
*stx1a/stx2c*	2	2	
*stx2g*	2		2
*stx1a/stx2* ^*a*^	1		1
*stx1a/stx2a*	1		1
*stx1c/stx2b*	1		1
*stx1c/stx2b*	1		1
*stx1c/stx2d*	1		1
*stx2d*	1		1

^a^subtype unknown

The concordance between agglutination and serogenotyping with regard to O-type was 83%. One isolate was assigned to different O-serotypes by the two methods. By serogenotyping and agglutination 12 and eight isolates respectively, were not assigned an O-serotype. The O-serogenotype was considered the true serotype when available. If an O-serogenotype was not available, the serotype revealed by agglutination was used. Serogenotyping always assigned an H-serotype.

**Fig. 1:**
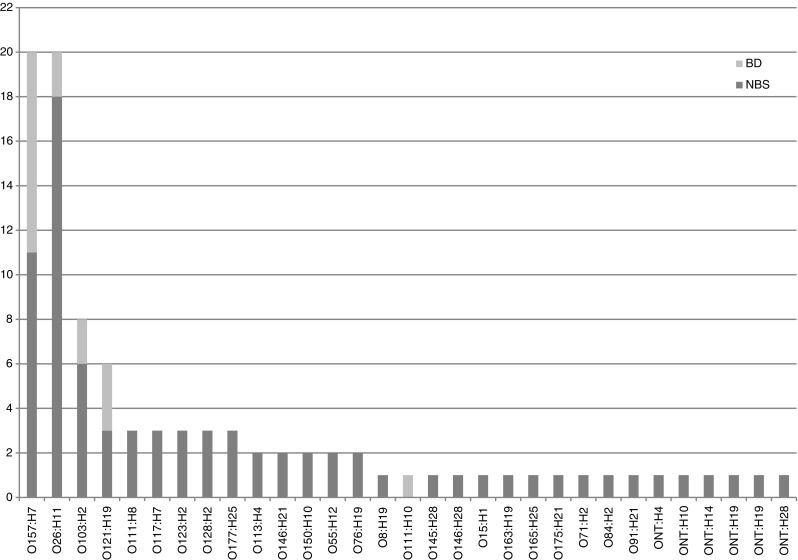
Distribution of serotypes among STEC causing BD and NBS, *ONT* O-serotype non-typeable

The most common serogenotypes were O26:H11 (*n* = 20) and O157:H7 (*n* = 20) followed by O103:H2 (*n* = 8) and O121:H19 (*n* = 6) (Fig. 1). Serogenotypes causing BD were O157:H7 (*n* = 9), O121:H19 (*n* = 3), O26:H11 (*n* = 2), O103:H2 (*n* = 2) and O111:H10 (*n* = 1), (Fig. 1).

### Microarray analysis

Analysis of the 96 virulence genes (including *stx*) in the microarray revealed that 62 genes (65%) were present in at least one isolate. Statistically significant differences (*χ*2 and Fisher’s exact test) in prevalence of these genes (Tables 3 and 4) were observed for:

- Twenty-one genes when comparing patients with BD and without bloody stool (18 of 21 associated with BD).
- Nine genes when comparing patients aged 0 to 4 to those aged 5 to 10 (six of nine associated with lower age).
- Two genes when comparing short (<3 weeks) to long (≥3 weeks) carriage (both genes associated with longer *stx* shedding).
- Sixteen genes when comparing low (0–3) to high (4–6) total score of clinical symptoms (ten of 16 associated with high score).

**Table 3:** Prevalence of genes where significant differences were observed in at least one of the comparisons (severity according to stool and age)

Gene	Severity according to stool					Age (years)				
	NBS		BD		*P*-value	0–4		5–10		*P*-value
	pos	prevalence	pos	prevalence		pos	prevalence	pos	prevalence	
eae	51	65%	16	94%	0.02^a^	50	74%	17	63%	0.31
efa1	36	46%	7	41%	0.74	36	53%	7	25%	0.012^a^
espB_O157	11	14%	9	53%	0.0012^a^	10	15%	10	36%	0.021^a^
espB_O26	34	44%	5	33%	0.44	34	52%	5	19%	0.0028^a^
lpfA	19	34%	0	0%	0.014^a^	14	30%	5	23%	0.54
cif	35	44%	4	24%	0.11	34	50%	5	18%	0.0036^a^
espA	44	61%	13	93%	0.029^a^	47	72%	10	48%	0.037^a^
espF	16	20%	9	53%	0.012^a^	13	19%	12	43%	0.016^a^
espF_O103H2	35	44%	7	41%	0.81	37	54%	5	18%	0.0010^a^
espJ	51	65%	16	94%	0.016^a^	50	74%	17	61%	0.21
etpD	10	13%	9	53%	0.015^a^	10	15%	9	32%	0.056
nleA	38	57%	11	92%	0.025^a^	42	69%	7	39%	0.021^a^
nleB	50	63%	16	94%	0.013^a^	49	72%	17	61%	0.28
nleB O157:	10	15%	6	50%	0.012^a^	13	22%	3	14%	0.45
nleC	37	47%	15	88%	0.0022^a^	38	57%	14	50%	0.55
tccP	42	53%	15	88%	0.0076^a^	41	60%	16	57%	0.76
astA	38	52%	15	88%	0.0063^a^	36	57%	17	63%	0.61
senB	7	9%	0	0%	0.35	3	4%	4	14%	0.19
toxB	12	17%	9	53%	0.0037^a^	13	21%	8	31%	0.33
stx1	53	68%	6	35%	0.012^a^	42	63%	17	61%	0.86
stx2	33	42%	13	76%	0.009^a^	30	44%	16	57%	0.25
stx2a	6	9%	9	53%	0.0001^a^	9	15%	6	23%	0.37
stx2c	9	13%	8	50%	0.0028^a^	10	17%	7	29%	0.23
ireA	7	9%	0	0%	0.34	2	3%	5	19%	0.02^a^
iss	38	48%	3	18%	0.021^a^	33	49%	8	29%	0.072
katP	36	46%	13	76%	0.021^a^	36	53%	13	46%	0.056
tir	50	63%	16	94%	0.013^a^	50	74%	16	57%	0.12

^a^ significant differences

**Table 4:** Prevalence of genes where significant differences were observed in at least one of the comparisons (length of shedding and symptom score)

Gene	Shedding (weeks)					Total clinical symptom score				
	>3		<3		*P*-value	0–4		5–10		*P*-value
	pos	prevalence	pos	prevalence		pos	prevalence	pos	prevalence	
eae	31	72%	21	68%	0.69	58	68%	9	90%	0.27
efa1	18	42%	12	39%	0.79	42	49%	1	10%	0.02^a^
espB_O157	8	19%	10	32%	0.18	13	15%	7	70%	0.0005^a^
espB_O26	19	45%	8	28%	0.13	39	47%	0	0%	0.0091^a^
lpfA	13	38%	3	14%	0.047^a^	18	30%	1	11%	0.43
cif	19	44%	11	35%	0.45	39	45%	0	0%	0.0005^a^
espA	26	68%	16	62%	0.57	51	65%	6	86%	0.42
espF	11	26%	11	35%	0.36	17	20%	8	80%	0.0003^a^
espF_O103H2	20	47%	10	32%	0.22	41	48%	1	10%	0.039^a^
espJ	31	72%	21	68%	0.69	58	67%	9	90%	0.27
etpD	8	19%	9	30%	0.26	12	14%	7	70%	0.0004^a^
nleA	22	63%	13	57%	0.63	44	60%	5	83%	0.4
nleB	30	70%	21	68%	0.85	57	66%	9	90%	0.16
nleB O157:H7	9	25%	6	25%	1.0	12	16%	4	57%	0.027^a^
nleC	22	51%	16	53%	0.86	44	52%	8	80%	0.11
tccP	27	63%	19	61%	0.90	48	56%	9	90%	0.045^a^
astA	23	56%	20	65%	0.47	44	55%	9	90%	0.043^a^
senB	6	14%	0	0%	0.04^a^	7	8%	0	0%	1.0
toxB	11	27%	8	30%	0.80	14	18%	7	70%	0.0013^a^
stx1	37	86%	15	50%	0.28	56	66%	3	30%	0.039^a^
stx2	21	49%	16	52%	0.81	38	44%	8	80%	0.045^a^
stx2a	7	18%	5	19%	1.0	10	13%	5	50%	0.013^a^
stx2c	10	24%	7	28%	0.74	11	15%	6	67%	0.017^a^
ireA	2	5%	2	6%	1.0	7	8%	0	0%	1.0
iss	17	40%	15	48%	0.45	41	48%	0	0%	0.0044^a^
kalP	21	49%	17	55%	0.61	42	49%	7	70%	0.32
tir	30	70%	21	68%	0.85	58	67%	8	80%	0.72

^a^ significant differences

### Duration of *stx* shedding in feces

The median length of carriage was 24 days (Table 1), which was used to separate short (<3 weeks) and long (≥3 weeks) carriage. Fisher’s exact test revealed two genes (*lpfA*, *senB*), associated with long duration of *stx* shedding in feces. Kaplan–Meier survival analysis (of the bacteria) was used to determine how the genetic makeup related to duration of shedding, and revealed seven genes influencing prolonged *stx* shedding; the presence of three genes (*lpfA*, *senB* and *stx1*) and the absence of four genes (*espB_O157*, *espF*, *astA* and *intI1*) predicted long duration of shedding.

### Multiple logistic regression

When comparing patients with BD and non-bloody stool, univariate logistic regression revealed statistically significant differences for the same genes as identified using the *χ*2 and Fisher’s exact test (Tables 3 and 4), with the exception of *lpfA*. The genes were assembled into the following groups: adhesins (*eae* and *espB_O157*), secretion systems (*espA*, *espF*, *espJ*, *etpD*, *nleA*, *nleB*, *nleB O157:H7*, *nleC*, *tccP*), toxins (*astA* and *toxB*), and shigatoxins (*stx1*, *stx2*, *stx2a*, *stx2c*) and analyzed separately using forward stepwise logistic regression. The univariate statistically significant genes from the miscellaneous group (*iss*, *katP* and *tir*) were not analyzed by multiple logistic regression due to their diverseness. *espB_O157*, *espF*, *nleC*, *astA*, *stx2a* and *stx2c* remained significant in the multiple logistic regression model. Of the eight genes (*espB_O157, espF*, *etpD*, *nleB O157:H7*, *stx2*, *stx2a*, *stx2c*) associated, by univariate logistic regression, with a high total score of clinical symptoms *espF* and *stx2a* remained significant in the multiple logistic regression model. When comparing patients of younger and older age, univariate logistic regression revealed statistically significant differences for the same nine genes as identified using the *χ*2 and Fisher’s exact test (Tables 3 and 4). Among these only *ireA* was significant in the multiple logistic regression model. A single gene (*lpfA*) was statistically significant in the univariate logistic regression model for duration of shedding; hence, no multiple logistic regression model was applied.

### Analysis of similarity

**Fig. 2:**
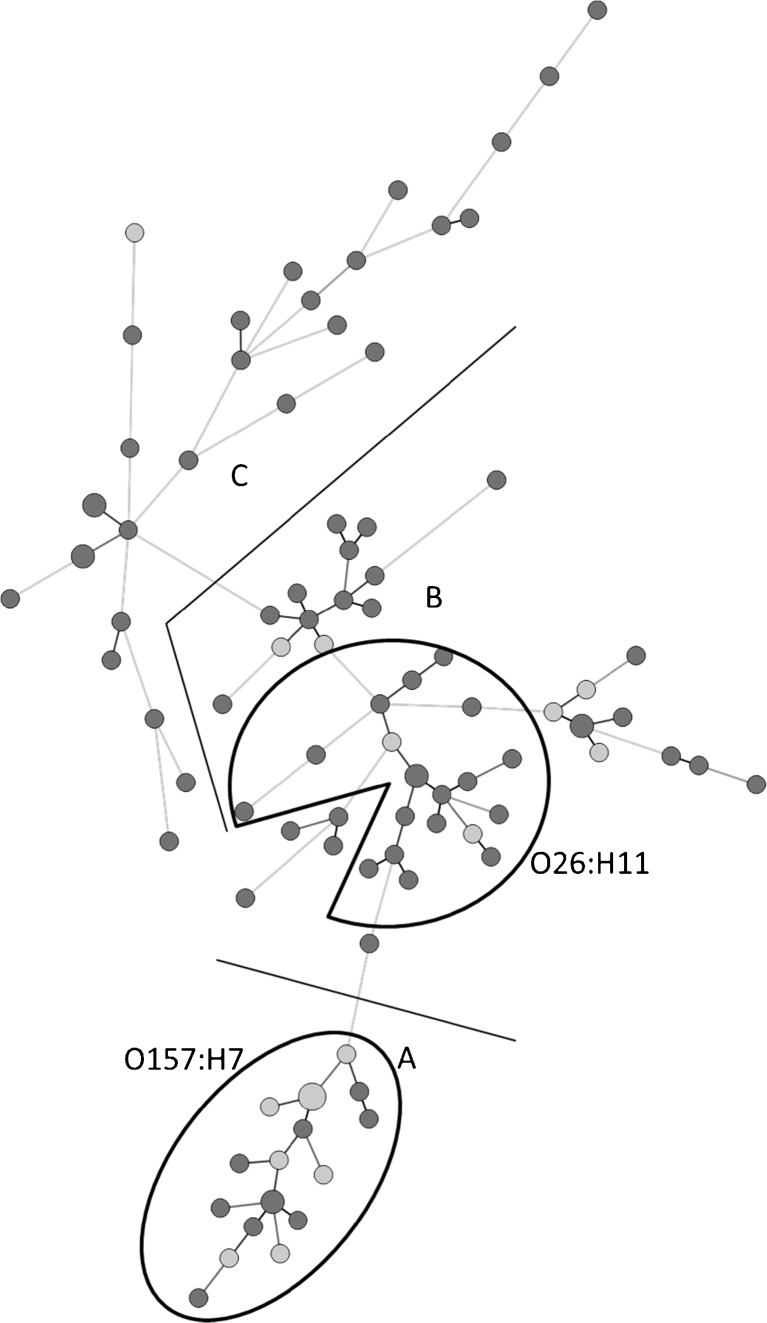
MST based on microarray results of 62 virulence genes for all STEC isolates. *eae* is present in all strains in groups A and B but in none of the strains in group C. In group A, all strains have an *stx2* subtype, and in group B 33% do. In group C, *stx1* dominates and 30% harbors *stx2b*. All strains with serotype O157:H7 are found in group A, and O26:H11 are found in group B. *Light grey* represents patients with BD and *dark grey* patients with NBS. The length of the branches is proportional to the distance between the types. The size of the nodes represents the number of isolates included in that type

**Fig. 3:**
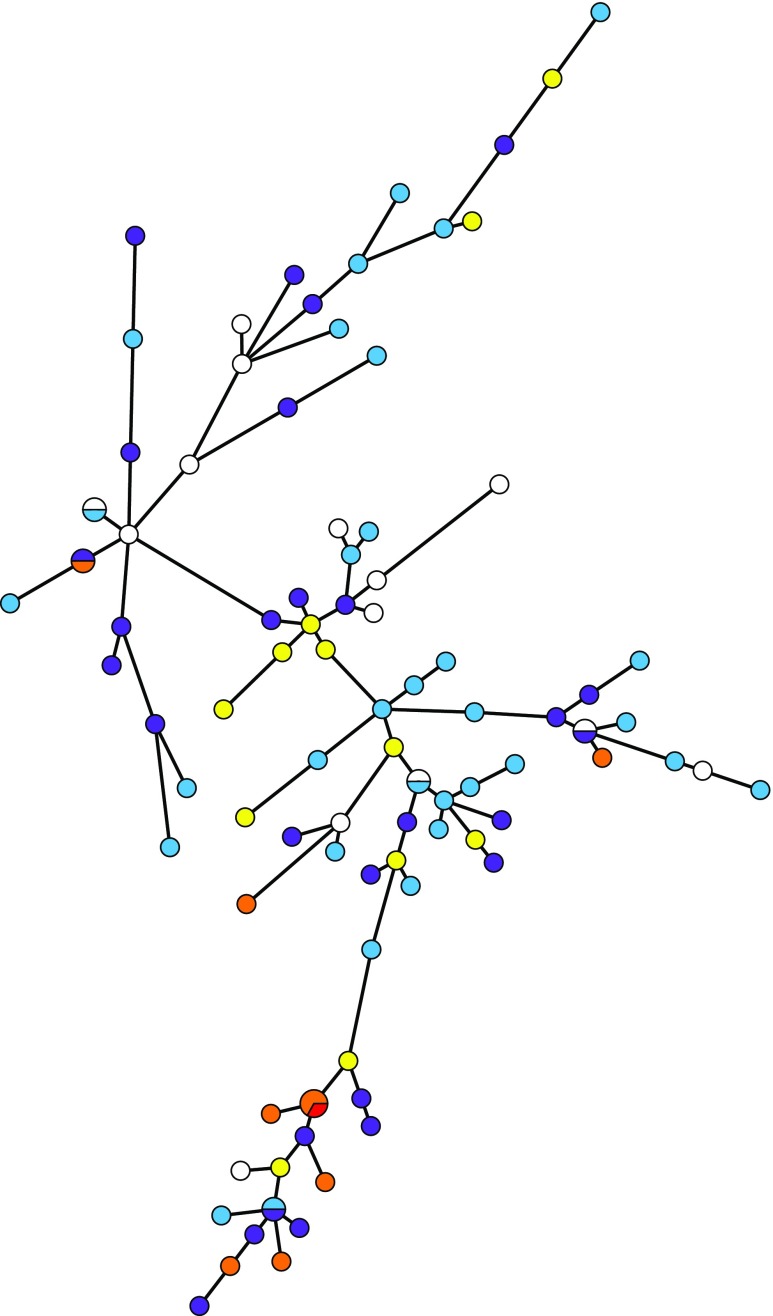
MST based on the microarray results of 62 virulence genes for all STEC isolates. *Dark blue* represents patients with a total clinical symptom score of 0, *light blue* = 1, *purple* = 2, *yellow* = 3, *orange* = 4 and *red* = 5. The length of the branches is proportional to the distance between the types. The size of the nodes represents the number of isolates included in that type

The analysis of similarity (MST) included the 62 virulence genes (including *stx*) present in at least one isolate. Serogenotypes and antibiotic resistance genes were excluded from the data used to construct the trees. To describe the isolates from the trees, the MSTs in Figs. 2 and 3 are divided into three groups (groups A–C, only shown in Fig. 2). In group A, all strains contained a *stx2* subtype and *eae*, in group B 33% contained *stx2* subtypes and all strains *eae*. In group C *stx1* dominated, 30% contained *stx2b*, and none of the strains contained *eae*. Group A was significantly associated with BD (*p* = 0.0004) and high symptom score (*p* = 0.0007).

### Antibiotic resistance gene content in STEC

Genes associated with resistance to aminoglycosides, beta lactams, chloramphenicol, macrolides, quinolones, sulphonamides, and trimethoprim were detected in low prevalence among the STEC isolates. The most common antibiotic resistance genes were *sul2*, *strB*, and *dfrA12* (present in 15, 14, and 13 isolates respectively, and encoding sulphonamide-, streptomycin- and trimethoprim resistance respectively).

## Discussion

In this study, we found various STEC genes associated with severe disease and long duration of *stx* shedding.

The microarray analyses revealed 62 virulence genes present in at least one of the isolates, and of these genes 18 were associated with severe disease (BD and one HUS case) and two genes with milder symptoms. Among the major virulence factors *stx2* and *eae* were associated with severe disease, which is in line with previous findings [27–29]. Among the severe STEC cases all carried subtype *stx2a* and/or *stx2c*, or alternatively, *stx1a*. Also multiple regression models showed that *stx2a* was associated with severe disease (BD and high total score of clinical symptoms). The subtypes *stx2a* or *stx2c* are known to cause severe outcome such as HC and HUS in STEC-infected patients [30]. In contrast, *stx1* and its subtypes have previously been associated with milder disease [12, 29, 31]. The MST cluster analysis showed three groups; A, B, and C (Fig. 2). Group A was significantly associated with BD; all of these strains contained *stx2* subtypes in combination with *eae*. This combination of virulence genes was also shown to be predictive for severe disease elsewhere [12]. Most strains in group B contained a subtype of *stx2*, and more than 60% of them harbored *eae*. In group C, all strains contained *stx1* or *stx2b*, and lacked *eae. stx1* subtypes and *stx2b* have only rarely been associated with severe disease in previous studies [12]. Our data indicate that there is a close genetic relationship between strains that are prone to cause BD and non-bloody stool (NBS). Recently, this has also been shown for EHEC O157 by typing based on 96 specific SNPs. Nine *E. coli* O157:H7 clades were defined, and clade 8 strains were associated with most cases of severe disease including HUS [18].

In addition to the major known virulence factors described above, 13 additional STEC genes were associated with BD, of which four (*espB_O157*, *espF*, *nleC*, *astA*) were still associated with BD after multiple logistic regression. Most genes encode toxins (e.g., *astA*, *toxB*), adhesion factors (i.e., *espB*_O157, *tir*) or secretion factors (e.g., *espA*, *espF*, *espJ*, *etpD*, *nleA*, *nleB*, *nleC*, *tccP*). The majority of these genes were also shown to be associated with BD in a recent Dutch study [15]. Furthermore, a recent diagnostic algorithm applied directly on fecal samples to assess the public health risk of STEC infection showed that six out of the 13 genes described above predict severity (*toxB*, *espA*, *tccP*, *nleA*, *nleB* and *tir*) [16]. In addition, Buvens et al. found that the individual genes *stx2*, *eae*, *espP*, *sen*, *nleB*, *nleE*, and the *efa* cluster were significantly more often present in non-O157 STEC associated with HUS [32]. STEC O157 is generally considered to cause more severe infections than non-O157 [33, 34]; this has, however, recently been questioned [35]. Also, our data, with severe cases (i.e., BD) caused by STEC of five different serotypes, indicate that serotype alone is not enough to predict how severe the infection will be. Instead, the results indicate a possibility of predicting disease severity based on STEC genetic makeup. In our study, we found two genes (*astA* and *katP*) associated with BD and one gene (*iss*) associated with NBS, which were not found in the studies by de Boer et al. and Ferdous et al. [15, 16].

The risk assessment of STEC virulence based on analysis of the genetic makeup of the actual strain might have the potential to provide valuable information to community health services in estimating the level of action required (with regard to source/contact tracing and infection control measures to minimize secondary transmission) to address the potential public health risk [30]. It has previously been described that rapid STEC detection is important in outbreak management and patient treatment [11]. In line with this, the microarray procedure can be performed in a single day, providing a potential tool for risk assessment. The clinical outcome in different patients infected by a certain STEC strain varies partly due to variation in, for example, age, immunity, ingested dose, and antimicrobial treatment. This may hamper the conclusions regarding virulence factors associated with disease severity. However, the statistical analysis and number of cases in this study makes the findings relevant, which are also in part described by others [15, 16].

Fisher’s exact test revealed two genes (*lpfA*, *senB*) associated with long duration of *stx* shedding in feces. *lpfA* encodes fimbriae, and *senB* encodes a toxin. In addition, Kaplan–Meier analyses revealed seven genes influencing prolonged shedding. Of these genes, the presence of three genes (*lpfA*, *senB*, and *stx1*) and the absence of four genes (*espB_O157*, *espF*, *astA*, and *intI1*) predicted long duration of *stx* shedding. Our data indicate that the presence of a combination of genes coding for fimbriae (*lpfA*) and two toxins (*senB* and *stx1*) and the absence of a toxin (*astA*), a secretion factor (*espF*), an adhesion molecule (*esp_O157*), and an integron (*intI1*), could predict long duration of *stx* shedding. It has to the best of our knowledge never been shown that the presence or absence of certain STEC virulence factors is associated with prolonged duration of *stx* shedding in humans. This could in future possibly be used to predict duration of shedding and to optimize, or individualize, infection control measures to minimize secondary transmission of STEC. Furthermore, a multiplex real-time PCR performed directly on feces to detect genes associated with long duration of STEC shedding could be developed based on these findings. However, the relevance of these genes in predicting long duration of shedding needs further investigation.

The distribution of serotypes in the present study was very similar to national as well as European STEC data (http://www.sva.se/globalassets/redesign2011/pdf/om_sva/publikationer/surveillance-2015-w.pdf, http://ecdc.europa.eu/en/publications/publications/food-and-waterborne-diseases-surveillance-report-2015.pdf). The concordance between traditional serotyping and serogenotyping was 83% for O-serotypes. Agglutination was not used to determine H-serotype. These combined data indicate that serogenotyping can be used to determine the serotype, which has also been shown in previous studies [25, 36].

In conclusion, we found STEC genes that might predict severe disease outcome already at diagnosis. This could perhaps be used to develop diagnostic tools for risk assessment of disease outcome and public health risk, including the food chain. Furthermore, genes associated with the duration of *stx* shedding were potentially detected enabling a possible better prediction of length of STEC carriage after infection.

## References

[1] Karmali MA, Steele BT, Petric M, Lim C (1983). Sporadic cases of haemolytic-uraemic syndrome associated with faecal cytotoxin and cytotoxin-producing Escherichia coli in stools. Lancet.

[2] O’Brien AO, Lively TA, Chen ME, Rothman SW, Formal SB (1983). Escherichia coli O157:H7 strains associated with haemorrhagic colitis in the United States produce a Shigella dysenteriae 1 (SHIGA) like cytotoxin. Lancet.

[3] Tarr PI, Gordon CA, Chandler WL (2005). Shiga-toxin-producing Escherichia coli and haemolytic uraemic syndrome. Lancet.

[4] Andreoli SP (2009). Acute kidney injury in children. Pediatr Nephrol.

[5] Hauswaldt S, Nitschke M, Sayk F, Solbach W, Knobloch JK (2013). Lessons learned from outbreaks of shiga toxin producing Escherichia coli. Curr Infect Dis Rep.

[6] Johnson KE, Thorpe CM, Sears CL (2006). The emerging clinical importance of non-O157 Shiga toxin-producing Escherichia coli. Clin Infect Dis.

[7] Buchholz U, Bernard H, Werber D, Bohmer MM, Remschmidt C, Wilking H, Delere Y, an der Heiden M, Adlhoch C, Dreesman J, Ehlers J, Ethelberg S, Faber M, Frank C, Fricke G, Greiner M, Hohle M, Ivarsson S, Jark U, Kirchner M, Koch J, Krause G, Luber P, Rosner B, Stark K, Kuhne M (2011). German outbreak of Escherichia coli O104:H4 associated with sprouts. N Engl J Med.

[8] Frank C, Werber D, Cramer JP, Askar M, Faber M, an der Heiden M, Bernard H, Fruth A, Prager R, Spode A, Wadl M, Zoufaly A, Jordan S, Kemper MJ, Follin P, Muller L, King LA, Rosner B, Buchholz U, Stark K, Krause G (2011). Epidemic profile of Shiga-toxin-producing Escherichia coli O104:H4 outbreak in Germany. N Engl J Med.

[9] Mellmann A, Harmsen D, Cummings CA, Zentz EB, Leopold SR, Rico A, Prior K, Szczepanowski R, Ji Y, Zhang W, McLaughlin SF, Henkhaus JK, Leopold B, Bielaszewska M, Prager R, Brzoska PM, Moore RL, Guenther S, Rothberg JM, Karch H (2011). Prospective genomic characterization of the German enterohemorrhagic Escherichia coli O104:H4 outbreak by rapid next generation sequencing technology. PLoS One.

[10] Gould LH, Bopp C, Strockbine N, Atkinson R, Baselski V, Body B, Carey R, Crandall C, Hurd S, Kaplan R, Neill M, Shea S, Somsel P, Tobin-D’Angelo M, Griffin PM, Gerner-Smidt P (2009). Recommendations for diagnosis of shiga toxin—producing Escherichia coli infections by clinical laboratories. MMWR Recomm Rep.

[11] Davis TK, McKee R, Schnadower D, Tarr PI (2013). Treatment of Shiga toxin-producing Escherichia coli infections. Infect Dis Clin North Am.

[12] Scheutz F, Teel LD, Beutin L, Pierard D, Buvens G, Karch H, Mellmann A, Caprioli A, Tozzoli R, Morabito S, Strockbine NA, Melton-Celsa AR, Sanchez M, Persson S, O’Brien AD (2012). Multicenter evaluation of a sequence-based protocol for subtyping Shiga toxins and standardizing Stx nomenclature. J Clin Microbiol.

[13] Wickham ME, Lupp C, Mascarenhas M, Vazquez A, Coombes BK, Brown NF, Coburn BA, Deng W, Puente JL, Karmali MA, Finlay BB (2006). Bacterial genetic determinants of non-O157 STEC outbreaks and hemolytic-uremic syndrome after infection. J Infect Dis.

[14] Monecke S, Mariani-Kurkdjian P, Bingen E, Weill FX, Baliere C, Slickers P, Ehricht R (2011). Presence of enterohemorrhagic Escherichia coli ST678/O104:H4 in France prior to 2011. Appl Environ Microbiol.

[15] Ferdous M, Friedrich AW, Grundmann H, de Boer RF, Croughs PD, Islam MA, den Bergh MF K-v, Kooistra-Smid AM, Rossen JW (2016). Molecular characterization and phylogeny of Shiga toxin-producing Escherichia coli isolates obtained from two Dutch regions using whole genome sequencing. Clin Microbiol Infect.

[16] de Boer RF, Ferdous M, Ott A, Scheper HR, Wisselink GJ, Heck ME, Rossen JW, Kooistra-Smid AM (2015). Assessing the public health risk of Shiga toxin-producing Escherichia coli by use of a rapid diagnostic screening algorithm. J Clin Microbiol.

[17] Karmali MA, Mascarenhas M, Shen S, Ziebell K, Johnson S, Reid-Smith R, Isaac-Renton J, Clark C, Rahn K, Kaper JB (2003). Association of genomic O island 122 of Escherichia coli EDL 933 with verocytotoxin-producing Escherichia coli seropathotypes that are linked to epidemic and/or serious disease. J Clin Microbiol.

[18] Manning SD, Motiwala AS, Springman AC, Qi W, Lacher DW, Ouellette LM, Mladonicky JM, Somsel P, Rudrik JT, Dietrich SE, Zhang W, Swaminathan B, Alland D, Whittam TS (2008). Variation in virulence among clades of Escherichia coli O157:H7 associated with disease outbreaks. Proc Natl Acad Sci U S A.

[19] Matussek A, Einemo IM, Jogenfors A, Lofdahl S, Lofgren S (2016). Shiga toxin-producing escherichia coli in diarrheal stool of Swedish children: evaluation of polymerase chain reaction screening and duration of shiga toxin shedding. J Pediatric Infect Dis Soc.

[20] Vonberg RP, Hohle M, Aepfelbacher M, Bange FC, Belmar Campos C, Claussen K, Christner M, Cramer JP, Haller H, Hornef M, Fickenscher H, Fraedrich K, Knobloch JK, Kuhbacher T, Manns MP, Nitschke M, Peters G, Pulz M, Rohde H, Roseland RT, Sayk F, Schaumburg F, Schocklmann HO, Schubert S, Solbach W, Karch H, Suerbaum S (2013). Duration of fecal shedding of Shiga toxin-producing Escherichia coli O104:H4 in patients infected during the 2011 outbreak in Germany: a multicenter study. Clin Infect Dis.

[21] Bryant PA, Venter D, Robins-Browne R, Curtis N (2004). Chips with everything: DNA microarrays in infectious diseases. Lancet Infect Dis.

[22] Geue L, Stieber B, Monecke S, Engelmann I, Gunzer F, Slickers P, Braun SD, Ehricht R (2014). Development of a rapid microarray-based DNA subtyping assay for the alleles of Shiga toxins 1 and 2 of Escherichia coli. J Clin Microbiol.

[23] Bellin T, Pulz M, Matussek A, Hempen HG, Gunzer F (2001). Rapid detection of enterohemorrhagic Escherichia coli by real-time PCR with fluorescent hybridization probes. J Clin Microbiol.

[24] Svenungsson B, Lagergren A, Ekwall E, Evengard B, Hedlund KO, Karnell A, Lofdahl S, Svensson L, Weintraub A (2000). Enteropathogens in adult patients with diarrhea and healthy control subjects: a 1-year prospective study in a Swedish clinic for infectious diseases. Clin Infect Dis.

[25] Geue L, Monecke S, Engelmann I, Braun S, Slickers P, Ehricht R (2014). Rapid microarray-based DNA genoserotyping of Escherichia coli. Microbiol Immunol.

[26] Geue L, Schares S, Mintel B, Conraths FJ, Muller E, Ehricht R (2010). Rapid microarray-based genotyping of enterohemorrhagic Escherichia coli serotype O156:H25/H-/Hnt isolates from cattle and clonal relationship analysis. Appl Environ Microbiol.

[27] Boerlin P, McEwen SA, Boerlin-Petzold F, Wilson JB, Johnson RP, Gyles CL (1999). Associations between virulence factors of Shiga toxin-producing Escherichia coli and disease in humans. J Clin Microbiol.

[28] Matussek A, Lauber J, Bergau A, Hansen W, Rohde M, Dittmar KE, Gunzer M, Mengel M, Gatzlaff P, Hartmann M, Buer J, Gunzer F (2003). Molecular and functional analysis of Shiga toxin-induced response patterns in human vascular endothelial cells. Blood.

[29] Proulx F, Seidman EG, Karpman D (2001). Pathogenesis of Shiga toxin-associated hemolytic uremic syndrome. Pediatr Res.

[30] Scheutz F (2014) Taxonomy meets public health: the case of shiga toxin-producing escherichia coli. Microbiol Spectr 2(3) 10.1128/microbiolspec.EHEC-0019-2013 26103973

[31] Gerber A, Karch H, Allerberger F, Verweyen HM, Zimmerhackl LB (2002). Clinical course and the role of shiga toxin-producing Escherichia coli infection in the hemolytic-uremic syndrome in pediatric patients, 1997–2000, in Germany and Austria: a prospective study. J Infect Dis.

[32] Buvens G, Pierard D (2012). Virulence profiling and disease association of verocytotoxin-producing Escherichia coli O157 and non-O157 isolates in Belgium. Foodborne Pathog Dis.

[33] Hadler JL, Clogher P, Hurd S, Phan Q, Mandour M, Bemis K, Marcus R (2011). Ten-year trends and risk factors for non-O157 Shiga toxin-producing Escherichia coli found through Shiga toxin testing, connecticut, 2000–2009. Clin Infect Dis.

[34] Hedican EB, Medus C, Besser JM, Juni BA, Koziol B, Taylor C, Smith KE (2009). Characteristics of O157 versus non-O157 Shiga toxin-producing Escherichia coli infections in Minnesota, 2000–2006. Clin Infect Dis.

[35] Preussel K, Hohle M, Stark K, Werber D (2013). Shiga toxin-producing Escherichia coli O157 is more likely to lead to hospitalization and death than non-O157 serogroups--except O104. PLoS One.

[36] Ballmer K, Korczak BM, Kuhnert P, Slickers P, Ehricht R, Hachler H (2007). Fast DNA serotyping of Escherichia coli by use of an oligonucleotide microarray. J Clin Microbiol.

